# The complete chloroplast genome of *Sparganium angustifolium* (Typhaceae)

**DOI:** 10.1080/23802359.2022.2135395

**Published:** 2022-10-27

**Authors:** Zhengying You, Wei Ming, Xiao Chang, Jie Li, Jia-nan Ying, Qixiang Lu

**Affiliations:** a College of Life Sciences and Medicine, Zhejiang Province Key Laboratory of Plant Secondary Metabolism and Regulation, Zhejiang Sci-Tech University, Hangzhou, China; b School of Mathematics and Statistics, Hubei Normal University, Huangshi, China

**Keywords:** Chloroplast genome, phylogenetic analysis, *Sparganium angustifolium*, Typhaceae

## Abstract

The complete chloroplast (cp) genome of *Sparganium angustifolium* was sequenced and annotated in the present study. The circular genome is 161,720 bp in length and exhibits a typical quadripartite structure with a large single-copy (LSC, 88,981 bp) and small single-copy (SSC, 18,731 bp) regions, separated by a pair of inverted repeats (IRs, 27,004 bp). The cp genome contains 114 unique genes, including 80 protein-coding, 30 tRNA, and four rRNA genes. The phylogenetic analysis of Typhaceae strongly supported the monophyly of *Sparganium* and resolved two clades that represented newly revised two subgenera. *S. angustifolia* has the closest relationship with *S. emersum* in the present sampling extent.

*Sparganium* (Typhaceae) is a marsh or aquatic genus including approximately 14 species distributed mainly in temperate and cool regions (Cook and Nicholls 1986, 1987; Kaul 2000). Species of *Sparganium* are ecologically important in aquatic communities by providing shelter and food for waterfowl and mammals (Fassett 1940). *S. angustifolium* Michaux 1803 is characterized by its long floating stems and leaves (>20 cm in length) different from its congeners (Sun and Simpson 2010), and shows a circumboreal distribution at high elevations. To date, the complete chloroplast (cp) genomes of five *Sparganium* species, *S. eurycarpum* subsp. *coreanum* (H.Lev.) C.D.K.Cook & M.S.Nicholls 1867, *S. stoloniferum* (Buch.-Ham. ex Graebn.) Buch.-Ham. ex Juz. 1934, *S. fallax* Graebn. 1900, *S. glomeratum* (Laest. ex Beurl.) Beurl. 1853 and *S. stoloniferum* subsp. *choui* (D.Yu) K.Sun 1992 have been reported (Gil et al. 2019; Su et al. 2019; Huang et al. 2021; Lu et al. 2021; Zhang et al. 2021). As genus *Sparganium* species are of ecological, phylogenetic and evolutionary value, more genetics resources would facilitate further study on them (Sulman et al. 2013). Here, we first reported the complete cp genome of *S. angustifolium* and reconstructed phylogenetic relationships within *Sparganium* and test the monophyly of this genus.

Fresh leaves of *S. angustifolium* were sampled from Hunchun city (130°48.480′E, 42°55.692′N) in Jilin Province of China, and dried with silica gel. The specimen was deposited in Herbarium of the Wuhan University (www.whu.edu.cn, Xinwei Xu, xuxw@whu.edu.cn) under the voucher number Xu3837. The plant material does not involve ethical conflicts. All the collection and following sequencing work was strictly executed under local legislation and related laboratory regulations to protect wild resources. Total genomic DNA was extracted using the DNA Plantzol Reagent (Invitrogen, Carlsbad, CA) following the manufacturer’s protocol. Library preparation and genomic DNA sequencing on the BGISEQ-500 platform were conducted by the Beijing Genomics Institute (BGI; Shenzhen, China). The obtained paired-end reads were used to assemble the cp genomes using NOVOPlasty4.2 (Dierckxsens et al. 2017), with a subunit of the photosystem II (*psb*A) gene from *S. stoloniferum* (GenBank accession no. NC_044634) as the seed. The cp genome annotation was performed in program Geneious Prime v2020.0.5 (Kearse et al. 2012), with the cp genome of *S. stoloniferum* as the reference. The start and stop codon positions and the boundaries between introns and exons were manually corrected where necessary. The annotated complete cp genomes of *S. angustifolium* were submitted to GenBank under the accession no. MZ981724.

The cp genome of *S. angustifolium* is a circular DNA molecule of 161,720 bp in length. The cp genome has a typical quadripartite structure, consisting of a pair of inverted repeats (IRa and IRb, 27,004 bp) separated by a large single-copy (LSC, 88,981 bp) region and a small single-copy (SSC, 18,731 bp) region. The overall GC content is 36.9%. The IR regions have a higher GC content (42.4%) than the LSC (34.8%) and SSC (31.0%). The cp genome encodes a set of 114 unique genes, including 80 protein-coding, 30 tRNA, and four rRNA genes. Among the genes identified, nine genes (*atp*F, *ndh*A, *ndh*B, *pet*B, *pet*D, *rpl*2, *rpl*16, *rpo*C1, and *rps*16) contain one intron and three genes (*clp*P, *rps*12, and *ycf*3) comprise of two introns. Twenty genes (eight protein-coding genes, eight tRNA genes, and four rRNA genes) are duplicated in the IR region, and *ycf*1 is pseudogene.

Chloroplast genomes of 15 Typhaceae species and two Bromeliaceae species were downloaded from GenBank for the phylogenetic analysis. A total of 18 sequences were aligned using the default settings in MAFFT v7.3 (Katoh and Standley 2013). The maximum-likelihood tree was constructed using IQTREE v1.6.7 with 1000 bootstrap replicates (Nguyen et al. 2015). *Ananas comosus* (L.) Merr. 1917 and *Tillandsia usneoides* (L.) L. 1762 were set as outgroups. The phylogenetic analysis provided strong support for monophyly of *Sparganium* (100% BS) and two main clades were recovered, which represented two *Sparganium* subgenera proposed by Sulman et al. (2013). In the present sampling extent, *S. angustifolia* has the closest relationship with *S. emersum* (Figure 1).

**Figure 1.**
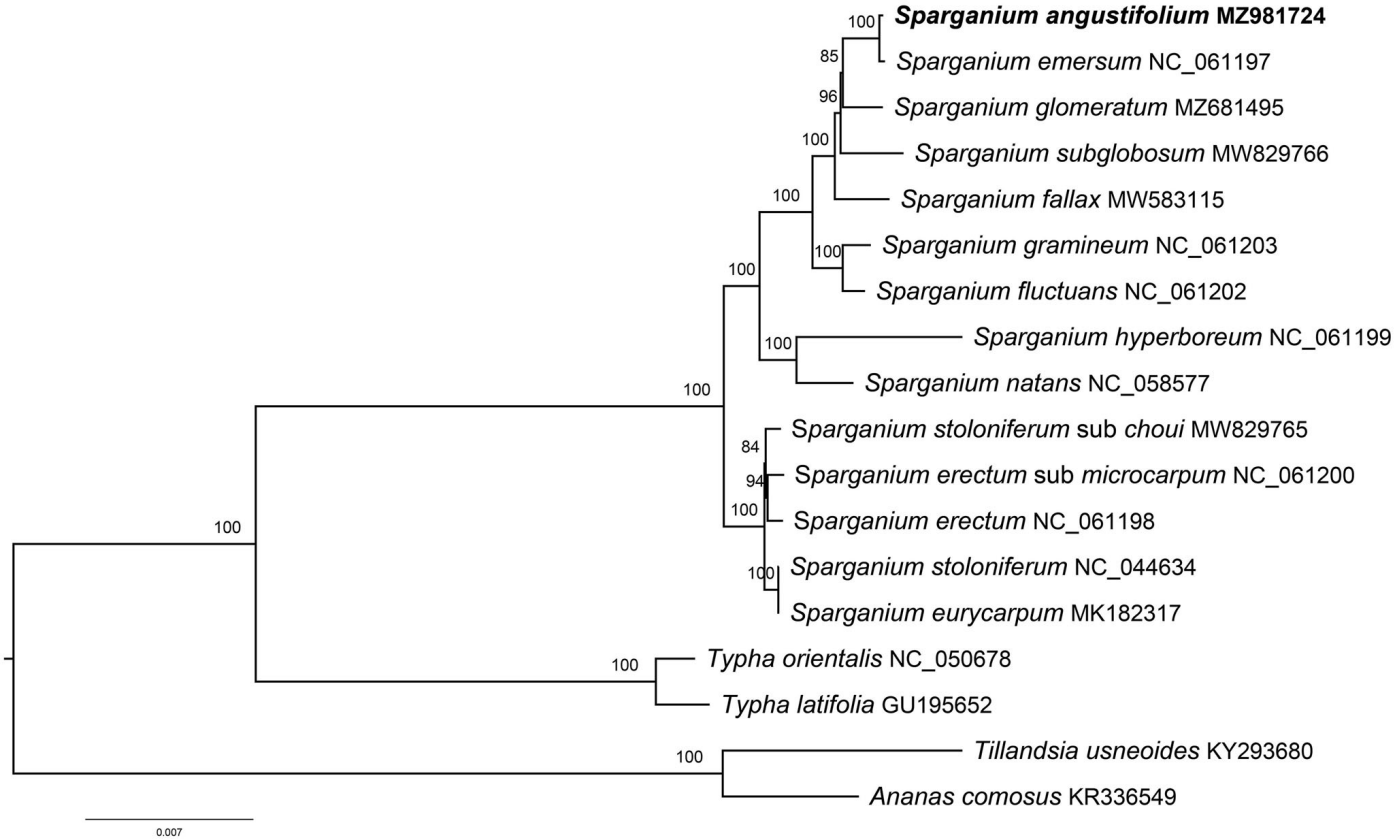
The maximum-likelihood tree inferred from 18 chloroplast genomes of Typhaceae and Bromeliaceae species (accession numbers are listed). The position of *Sparganium angustifolium* is highlighted in bold and numbers above each node are bootstrap support values.

## Author contributions

QL and ZY conceived the idea; QL contributed to the sampling; XC, JL, and JY collected the data; WM analyzed the data. The manuscript was written by ZY and WM. All authors read and approved the final manuscript.

## Data Availability

The genome sequence data that support the findings of this study are openly available in GenBank of NCBI at https://www.ncbi.nlm.nih.gov/ under the accession no. MZ981724. The associated BioProject, SRA, and Bio-Sample numbers are PRJNA750266, SRR16530833, and SAMN20691739, respectively.
